# Comparison of Behavioral and Sexual Problems between Intellectually Disabled and Normal Adolescent Boys during Puberty in Yazd, Iran

**Published:** 2014

**Authors:** Leila Akrami, Maryam Davudi

**Affiliations:** 1 MSc, PhD Student, Department of Psychology of Exceptional Children, School of Psychological and Educational Sciences, University of Isfahan, Isfahan, Iran.; 2 Department of Psychological and Educational, Islamic Azad University, Abarkooh Branch, Yazd, Iran.

**Keywords:** Adolescence, Behavioral and Sexual Problems, Intellectual Disability, Puberty

## Abstract

**Objective:** To compare sexual and behavioral puberty problems between intellectually disabled (ID) and normal boys in Yazd, Iran.

**Methods:** In the present study, 65 intellectually disabled and 65 normal boys were included. The Child Behavior Check List (CBCL) was used to investigate behavioral problems. In order to study sexual problems, a questionnaire that was designed by the researchers was applie.

**Results: **Anxiety, depression, social problems, attention problems, aggressiveness, and sexual problems were more frequent in intellectually disabled boys than in normal boys. On the other hand, regarding somatic complaints, withdrawal, thought problems, internalizing, delinquent behavior, and externalizing there was no difference between the two groups.

**Conclusion:** Behavioral and sexual problems are more common in adolescent boys with intellectual disability (ID) than in normal boys during the puberty period. Therefore, puberty is an important period for intellectually disabled boys and their families; this should be taken into consideration by psychologists and clinicians.

## Introduction

Puberty is the first stage of adolescence and is one of the possible challenges of this period. Puberty is a process of physical changes and its main characteristic is the development of instinct and secondary sexual traits. Puberty is the origin of extensive changes, and its somatic changes can affect the mental, behavioral, and social performances of an individual (1).

Boys with intellectual disability (ID) have patterns of genital development similar to other boys (2). Puberty is an important period for boys and girls with ID. It is important to understand how boys with ID act during puberty and grow up. This understanding combined with the awareness of parents about what teaching approaches work best can prepare their children for puberty (3).

During adolescence and puberty, the most important mental disorders in individuals with ID are mood and behavioral disorders. Mood disorders in intellectually and developmentally disabled and normal adolescents are similar. Among the common symptoms, one may refer to anxiety, phobia, aggressiveness, obsessions, attention deficit disorder, and somatic diseases. The early symptoms usually consist of different changes in vital body functions such as losing appetite, insomnia, and etcetera. External stressful events are often found to be the cause, but ordinary lifestyle changes may also be responsible (4). The other mood disorders include severe depression, social withdrawal, and recurrence of some behaviors related to the previous life periods (5).

Bradley et al. believe that psychotic and behavioral disorders generally increase by about 3% to 4% after puberty in adolescents with ID (6). The results of the studies by Dekker et al. and Liu et al. showed that problems such as anxiety, depression, and somatic complaints in the trainable and educable mentally-retarded individuals are stronger than in individuals with ID. Attention problems, aggression, thought problems, and withdrawal were more prevalent in both groups than in the normal population (7, 8).

Studies show that adolescents with intellectual and developmental disability have extended anxiety disorders during puberty (3).

In 1996, Jafarinezhad conducted an investigation to compare social puberty in normal students and students with ID in Iran. The Vineland Adaptive Behavior Scale (VABS) was used to assess the students' social puberty. The results suggested a significant difference between the averages of these two groups. This difference indicates a considerable weakness in the social puberty of students with ID as compared to the social puberty of normal students (9).

Many people wrongly imagine that having a low IQ, a child with ID must be less active in other fields as well, while sometimes the reverse is true. It is because sexual stimulations and behavior have nothing to do with the mind and are guided by the endocrine hormones. Since children with ID are less aware of social rules, these sexual desires may arise sooner in them than in others (10).

Numerous studies show that the knowledge about sexual issues in individuals with ID is lower than that in normal people. These individuals cannot obtain this knowledge from others. They are not able to read and comprehend books and magazines as sources of information in this regard. Usually, in their schools, there is not enough opportunity for teaching these matters as well as features of their own sex and distinctions between the two sexes. We need to consider that individuals with ID are more exposed to sexual abuse than are normal people. Different studies indicate that sometimes early maturation in intellectually disabled individuals and the quick changes related to it can increase sexual perversions and abuses (11).

Previous studies showed that sexual problems and severe sexuality (for example sexuality to opposite sex, sexual gaudiness, and etcetera) are stronger in individuals with ID at home, school, on the street, and etcetera (12-16). Most findings in recent years show an increase in behavioral and psychological disorders in adolescents with ID. It should be noticed that by reaching the age of puberty, adolescents seriously begin to experience sexual attraction and become sensitive to sexual subjects. Indeed, their thoughts become preoccupied with sexual matters. In many cases, sexual perversions appear as fake satisfactions. These fake satisfactions are actually deviations from the right path of sexual satisfaction, something viewed as sexual perversion manifested in different forms such as masturbation, illegal relationships, and etcetra (17).

There is limited knowledge about sexual issues in boys with ID. Unfortunately few studies have investigated puberty problems (sexual and behavioral problems) of individuals with ID. Research of puberty is important for boys with ID and will increase awareness of parents to prepare their children for it (2). In some countries (for example Iran) it is difficult to find resources that are understandable, realistic, and concrete enough to explain the teaching requirements of boys with ID and their families during puberty. Usually, people with ID receive too little information about changes during puberty, and, therefore, they have sexual and behavioral problems. The present study investigates the puberty problems (sexual and behavioral problems) in normal boys and boys with ID in Yazd, Iran.

## Materials and Methods

***Design and setting***

This descriptive study was carried out in 2009 in Yazd, Iran.

***Participants and sampling***

The participants were chosen from ordinary and exceptional public schools; 65 boys with ID and 65 normal boys were selected via multi-level cluster random sampling. 

Inclusion criteria of each group consisted of intellectually disabled boys with IQ = 50-70 (i.e., educable ID), and boys with ID and normal boys at the age of 12-16 who had not experienced precocious puberty (puberty occurring at an unusually early age).

The questionnaires were given to the students' mothers after explanation about objectives and advantages of the research and the method of filling the questionnaires by educators. For justification, gaining consent, and safety of the students' mothers, we asked the mothers not to write down the name of their child.

The study protocol was in accordance with the standards of the ethics committee.

***Measures***

The instruments used in this study were a checklist and one questionnaire.

*Child Behavior Checklist (CBCL)*

This checklist contains 113 items and the parents filled out this checklist. The scoring was done after administrating the test according to CBCL's instructions for the 4-18 year olds. Generally, children’s behavioral and affective signals in CBCL are reflected in 8 subscales. However, withdrawal, somatic complaints, anxiety and depression, social problems, attention problems, delinquent, and aggressive behavior were added to these 8 subscales. Then, the scores were calculated on each scale, internalizing problems (withdrawal, somatic complaints, and anxiety and depression), and externalizing problems (aggressiveness and delinquency). Finally, the total scores of the test were reported (18).

The reliability coefficient of the CBCL calculated by Achenbach with Australia's common population samples was r = 0.87 (16). It is to be mentioned that CBCL is used for exceptional children frequently. In an investigation, Embregts assessed the reliability of CBCL for the intellectually disabled group. The Pearson coefficient obtained through test-retest was r = 0.89, and the obtained Cronbach's Alpha was 0.92 (18, 19).

The Persian version of CBCL has been prepared by Tehrani-Doost et al. These results support the multicultural CBCL/TRF findings. CBCL is a useful instrument to study ADHD and any disorders in community samples (20).

*Sexual problems questionnaire*

To assess boys’ (both normal and with ID) sexual problems during the puberty period, a questionnaire designed by the researcher was used. After defining the variables and the following sources in study field, the questionnaire was provided. 

The questionnaire had 2 sections. The first section asked for personal information such as the adolescent’s age, the family’s income, the average of educational scores, the number of family members, and the mother's education level. The second section contained 24 multiple-choice questions that investigated the sexual problems in the two groups (with ID and normal). The questionnaires were completed by the participants’ mothers. The answers were registered by two words, Yes or No, and were scored 0 and 1, respectively. To give honest responses, the mothers were asked not to write their names and their children's names. All of the questions were compared between the two groups using chi-squared test. 

To calculate content-related validity, the questionnaire items were examined and judged by some professors of different fields of study in different universities. After collecting all the opinions, the consistency (agreement) coefficient of 0.71 was obtained, and then the necessary corrective changes were made to make the questionnaire ready for data collection. To calculate (nonparametric) the Spearman correlation coefficient, a test-retest method was applied within a 2-week interval for both groups (with ID and normal boys) with 12 subjects in each group. The coefficient obtained was r = 0.75 for normal boys which is significant at α = 5% level and r = 0.74 for boys with ID which is significant at α = 5% level. Moreover, Kuder–Richardson's (KR) method was used to determine the internal isotropy of the questionnaire. Using KR_21 _coefficient, the obtained coefficient for boys with ID was 0.75 in the first test and 0.79 in the second test. For the normal boys, KR_21_ was 0.72 and 0.73 in the first and second tests, respectively.

***Statistical analysis***

Once the questionnaires were gathered, the collected data were analyzed by means of statistical methods. Analyses were performed using SPSS for Windows (version 12.0; SPSS Inc., Chicago, IL, USA). For the analyses, statistical tests such as Mann-Whitney, t-test, and chi-squared were used. P values less than 0.05 were considered significant.

## Results

Table 1 presents the demographic profile of 130 subjects. About 40% of the subjects were boys with ID and 38% normal boys at the age of 13. About 35.38% of the mothers of normal boys and 43.07% of the mothers of the ID group had a high school diploma. 

About 46.16% of boys with ID and 49.24% of normal boys were from middle economic class based on the family’s income. Range of age between 14-16 was observed in about 41.53% of boys with ID and 32.30% of normal boys.

***Behavioral problems***

Tables 2 and 3 present behavioral problems in both with ID and normal boys. The result indicated that, statistically, no significant difference was observed between the two groups regarding withdrawal, somatic complaints, thought problems, delinquent behavior, externalizing, and internalizing.

*Sexual problems*

As the results indicate, some sexual problems, such as sexual gaudiness, masturbation in public, and severe sexuality toward the opposite sex, were more prominent in boys with ID than normal boys. About 7.69% of boys with ID were found to have experience of sexual abuse (Table 4).

***Total scores of sexual and behavioral problems***

Table 5 demonstrates that there was a significant difference between the total scores of behavioral and sexual problems between the two groups. Boys with ID had more behavioral problems (p = 0.003) and more sexual problems (p = 0.004) than normal boys.

**Table 1:** Demographic profile of the samples (n = 130 boys**)**

**Age (years)**	**Normal boys (65 subjects)**	**Intellectually disabled boys (65 subjects)**	**Education of mothers**	**Normal boys**	**Intellectuallydisabled boys**
**12**	05 (7.69%)	6 (9.23%)	Higher than high school diploma	22 (33.84%)	24 (36.92%)
**13**	25 (38%)	26 (40%)	High school diploma	23 (35.38%)	28 (43.07%)
**14**	15 (23.07%)	12 (18.46%)	Primary school	07 (10.76%)	09 (13.84%)
**15**	13 (20%)	14 (21.53%)	Guidance school	13 (20%)	04 (6.15%)
**16**	07 (10.79%)	7 (10. 76%)	Illiterate	0	0

**Table 2:** Comparison of behavioral problems in intellectually disabled versus normal adolescent boys in Yazd, Iran

	**Normal boys**	**Intellectually disabled boys**	**Mann- Whitney**	**P-value**
**Withdrawal**	62.07 (± 17.01)	75.83 (± 18.11)	2.15	0.140
**Somatic complaint**	42.16 (± 10.50)	38.61 (± 9.73)	0.56	0.510
**Thought problem**	43.71 (± 12.13)	47.37 (± 10.83)	1.77	0.550
**Delinquent behavior**	57.81 (± 13.44)	53.22 (± 11.09)	1.71	0.620
**Internalizing**	30.12 (± 6.02)	32.20 (± 8.29)	1.58	0.072

**Table 3:** Comparison of behavioral problems between intellectually disabled and normal adolescent boys in Yazd, Iran

	**Normal boys**	**Intellectually disabled boys**	**Student’s t- test**	**P-value**
**Anxiety/Depression**	3.12 (± 1.70)	6.23 (± 3.45)	1.08	0.002
**Attention disorder**	4.30 (± 2.23)	5.76 (± 2.61)	2.14	0.013
**Aggressiveness**	3.57 (± 6.12)	6.12 (± 2.18)	3.11	0.002
**Externalizing**	3.54 (± 3.23)	3.23 (± 1.53)	2.08	0.310
**Social problems**	6.44 (± 8.17)	8.17 (± 3.51)	2.11	0.002

**Table 4:** Comparing frequency (percentage) of sexual problems between intellectually disabled and normal adolescent boys in Yazd, Iran

	**Normal boys**	**Intellectually disabled boys**	**Chi- squared test**	**P-value**
**Sexual gaudiness**	02 (3.07%)	27 (41.53%)	32.481	< 0.001
**Gender identity disorder**	04 (6.15%)	3 (4.61%)	00.157	< 0.685
**Exhibitionism**	0	3 (4.61%)	03.093	< 0.078
**Homosexuality**	04 (6.15%)	0	04.187	< 0.410
**Masturbation in public**	0	17 (26.5%)	20.48	< 0.001
**Masturbation in private**	03 (4.61%)	8 (12.3%)	04.766	< 0.230
**Severe sexuality toward opposite sex**	02 (3.07%)	29 (44.61%)	34.081	< 0.001
**Voyeurism**	11 (16.92%)	4 (6.15%)	03.950	< 0.058
**Sexual abuse**	0	5 (7.69%)	04.053	< 0.513

**Table 5:** Comparison of mean (± SD) of total scores of sexual and behavioral problems in intellectually disabled and normal adolescent boys in Yazd, Iran

	**Normal boys**	**Intellectually disabled boys**	**Student’s t- test**	**P-value**
**Total score of behavioral problems**	42.12 (± 18.43)	51.32 (± 19.22)	3.11	0.003
**Total score of sexual problems**	0.18 (± 0.13)	0.28 (± 0.15)	2.21	0.004

## Discussion

During the puberty period, the rate of sexual and behavioral problems increases. 

The results show that there is a significant difference between the average scores on the behavioral problems in boys with ID and normal boys. This finding is in agreement with the results achieved by previous studies (6-9).

The findings of the present research may be explained in that behavioral problems are observed more frequently in children and adolescents with ID than in individuals with normal intelligence. One must consider that low intelligence is accompanied with affective, insufficiency, personality problems, and behavioral disorders (21). Besides, social environments such as family, school, and etcetera with unsuitable communications can cause behavioral disorders to appear. Therefore, the above factors bringing about different changes in adolescence period can initiate and intensify behavioral problems in intellectually disabled individuals (22).

Unsuitable behaviors are caused by low intelligence, stimulation, and personality factors. The majority of sexual and somatic changes show up in aggressive patterns of behavior during adolescence period. This aggressiveness may increase even further later in their life. Aggressiveness is often practiced by groups of the same age, who aggravate and intensify it in each other. A mentally retarded child is a person who expects fewer successes and accepts more failures. Perhaps these expectations arise from experiencing sequential failures in doing difficult jobs. Therefore, the child reacts to the failures in inappropriate ways. Aggressive behavior in adolescents with ID arises from lack of development in moralities. A low level of moral argumentation in these children makes them aggressive and offensive (23).

The results show that there is a difference between the average of sexual problems in boys with ID and in normal boys. This finding is in accordance with the results achieved from former studies (12-16).

People with ID experience the same range of sexual needs and desires as other people do. However, they may not be able to communicate or act on these desires and may struggle with learning appropriate sexual behavior. They may also be hampered by the attitudes of other people; beliefs persist in the society that people with intellectual disability are childish (24).

***Limitations***

Because of intellectually disabled boys’ incapacity to respond, their mothers were asked to fill out the checklist. It is possible that some mothers may not have been truthful in their answers to the sexual problems questionnaire because of cultural problems in Iran, especially mothers of normal boys.

In summary, this paper tried to investigate behavioral and sexual problems in boys with ID during puberty period. Research about behavioral and sexual problems during puberty period in girls with ID is also necessary. Since the present research has merely focused on the problems of the youth in 12-16 year olds, the literature calls for other studies to aim at behavioral disorders in other periods of life. 

## Footenote

**Declaration of interest:** None.
